# Gestational Trophoblastic Disease in Brazil

**DOI:** 10.1055/s-0039-1688566

**Published:** 2019-04

**Authors:** Antonio Braga, Lawrence Hsu Lin, Izildinha Maestá, Sue Yazaki Sun, Elza Uberti, José Mauro Madi, Maurício Viggiano

**Affiliations:** 1 Department of Obstetrics and Gynecology, Universidade Federal do Rio de Janeiro, Rio de Janeiro, RJ, Brazil; 2 Department of Obstetrics and Gynecology, Universidade de São Paulo, São Paulo, SP, Brazil; 3 Department of Obstetrics and Gynecology, Universidade Estadual Paulista, Botucatu, SP, Brazil; 4 Department of Obstetrics, Paulista Medical School, Universidade Federal de São Paulo, São Paulo, SP, Brazil; 5 Maternity Mario Totta, Santa Casa de Misericórdia, Porto Alegre, RS, Brazil; 6 Center for Life Science, Universidade de Caxias do Sul, Caxias do Sul, RS, Brazil; 7 Department of Obstetrics and Gynecology, Universidade Federal de Goiás, Goiânia, GO, Brazil

Gestational trophoblastic disease (GTD) is a group of conditions characterized by abnormal proliferation of placental trophoblast. Hydatidiform mole (HM) is the most common form of GTD, which has a frequency of 1 case per 1,000 pregnancies in North America and Europe; however, the incidence of the disease is thought to be at least two to three times higher in Brazil.^1^ ^2^

In developing countries, due to the delay in diagnosis of HM, it is not uncommon for patients to develop clinical complications, which can represent causes of potentially life-threatening conditions, maternal near miss and maternal deaths.^3^ The only way to prevent those adverse outcomes is the early diagnosis of HM and prompt uterine evacuation. After uterine evacuation, patients with HM need to be carefully followed due to the risk of development of gestational trophoblastic neoplasia (GTN). The early diagnosis of GTN is key to ensure cure, and patients with late diagnosis of GTN require more aggressive and toxic treatment and hold worse prognosis, often with metastatic disease.^1^ Official national data on the morbidity and mortality rates of patients with GTD is not available. Currently, a nationwide collaborative study is in progress to better understand the morbidity and mortality of patients with GTN in Brazil, through the Brazilian Network for Gestational Trophoblastic Disease Study Group, which has developed several collaborative studies over the last years to advance the understanding of GTD.^4^ ^5^

The Comissão Nacional Especializada em DTG (Special National Committee on GTD), an initiative of the Federation of Brazilian Associations of Gynecology and Obstetrics (FEBRASGO), has been focusing on continued medical education through free online management protocols and several national and regional teaching events with the Nation's leading specialists.

The importance of reference centers in the management of patients with GTD has been highlighted in various studies, showing that patients present better outcomes when they are initially followed at those centers.^6^ The GTD reference centers are able to follow and manage patients within the full spectrum of GTD with a multidisciplinary team and a personalized approach. The Associação Brasileira de Doença Trofoblástica Gestacional (Brazilian Association of Gestational Trophoblastic Disease), over the last years, had the task to help to establish GTD reference centers across the Nation. Currently, there are 47 centers in Brazil and at least one in each of the Brazilian states, shortening the distance between the patients and the treatment of excellence through the public health system.^7^

The official government referral system that refers patients in primary care to secondary and tertiary care is one way for patients to reach a GTD reference center. However, this referral system is far from being perfect, as many patients are referred late, sometimes with severe complications. Therefore, making technology and social media allies, the Associação Brasileira de Doença Trofoblástica Gestacional created a Facebook page (currently with over 7,700 members, including patients, relatives and healthcare providers) that helps patients reach the nearest reference center, find reliable information of the disease online, and also serve as a patient support group.^8^ ^9^

Gestational trophoblastic disease is a complex group of diseases. Their early diagnosis and treatment are fundamental to achieve cure.^10^ Brazil, being a country of continental dimensions, makes the task of treating patients with such rare diseases very difficult. Therefore, several initiatives by Associação Brasileira de Doença Trofoblástica Gestacional and Comissão Nacional Especializada em DTG are in action and being implemented to foster the better care of patients with GTD across the Nation.
